# Training the component steps of an extra-corporeal membrane oxygenation (ECMO) cannulation outside the clinical setting

**DOI:** 10.1007/s10047-020-01176-x

**Published:** 2020-06-08

**Authors:** Sanne M. B. I. Botden, Guus M. Bökkerink, Erik Leijte, Tim Antonius, Ivo de Blaauw

**Affiliations:** 1 Department of Pediatric Surgery, Radboudumc – Amalia Children’s Hospital, Route 618, PO Box 9101, 6500 HB Nijmegen, The Netherlands; 2 Department of Pediatric Surgery, Princess Maxima Center, Utrecht, The Netherlands; 3 Department of Neonatology, Radboudumc – Amalia Children’s Hospital, Nijmegen, The Netherlands

**Keywords:** Extra-corporeal membrane oxygenation, Training, Simulation, Artificial model

## Abstract

Extra-corporeal membrane oxygenation (ECMO) cannulation can be a stressful procedure because a fast cannulation is vital for the patient’s survival. Therefore, it is important to train the steps of cannulation outside the clinical setting. A relatively low budget, easy to use model, was developed to train the most important steps of an ECMO cannulation. Following this, it was evaluated by experts and target group participants. They all completed a questionnaire regarding their experience and opinions on the ECMO model on general aspects and the training of the component steps, rated on a 5-point Likert scale. Twenty-one participants completed the questionnaire. The features and steps of the model were rated with a mean of 3.9 on average. The haptics of the landscape scored least, with a mean of 3.6, although the haptics of the vessels scored highest with 4.0. The rating of the component steps showed that only ‘opening of the vessels’ was scored significantly different between the expertise levels (means experts: 4.0, target group: 3.4, *p* = 0.032). This low budget model is considered to be a valid tool to train the component steps of the ECMO cannulation, which could reduce the learning curve in the a stressful clinical setting. Level of evidence: II prospective comparative study.

## Introduction

Extra-corporeal membrane oxygenation (ECMO) is an intervention carried out on patients who are in a life-threatening situation due to pulmonary and/or cardiac failure. The initiation of ECMO is time critical, as is the ongoing management of these patients, because any issue during the therapy can irreversibly compromise the patient’s outcome. The use of ECMO in children and neonates is limited to centers which have the opportunity to perform this treatment. Fortunately, ECMO is not necessary in pediatric patients very often, although this makes it a relatively rare procedure for pediatric (cardiac) surgeons, which has to be performed during a resuscitation and sometimes even reanimation setting.

The cannulation of an unstable neonate or infant on extra-corporeal membrane oxygenation (ECMO) can be a stressful procedure for a surgeon. Although the dissection of the vessels is not considered complex, the insertion and fixation of the cannulas should be performed following a particular protocol and systematically to ensure a successful cannulation and ECMO run. Problems or delays in successful cannulation can result in the loss of a patient. Therefore, it is important to have the opportunity to train these steps outside the clinical setting.

Simulation-based education (SBE), from the simplest approaches to the most immersive modalities, helps promote optimal individual and team performance [1]. There are training programs for ECMO cannulation; however, these are often based on the ECMO system and pathophysiology that has to be overcome with the ECMO treatment [1–4]. Although these team trainings and system simulations are extremely important, the focus of this research is the training of the cannulation of the ECMO cannulas in the vessels because this is of paramount importance in the ECMO treatment.

The use of a cannulation model has been described before, only in these models the authors mainly focus on adults and punctured guided cannulation [3, 5]. Other methods to practice the cannulation is the use of animal models [6], which is expensive and not easily accessible for most surgeons, runs against the aim to reduce the use of animal models. Although there are a few models developed for the training of ECMO cannulation in neonatal and pediatric patients, these are relatively expensive, not readily available and therefore not suitable for routine use to practice cannulation [7].

We present a low budget training model for the cannulation of ECMO cannulas in neonates and infants, to train the component steps of the cannulation pediatric (cardiac) surgical trainees in any preclinical setting.

## Methods

### Development of the ECMO model

To achieve training in the component steps of the cannulation, the aim of this research was to develop a 3-Dimensional model resembling the carotid artery and jugular vein which could be cannulated to an ECMO system. Other important features of the model had to be low cost, reusable and present the opportunity to simulate blood flow.

**Fig. 1:**
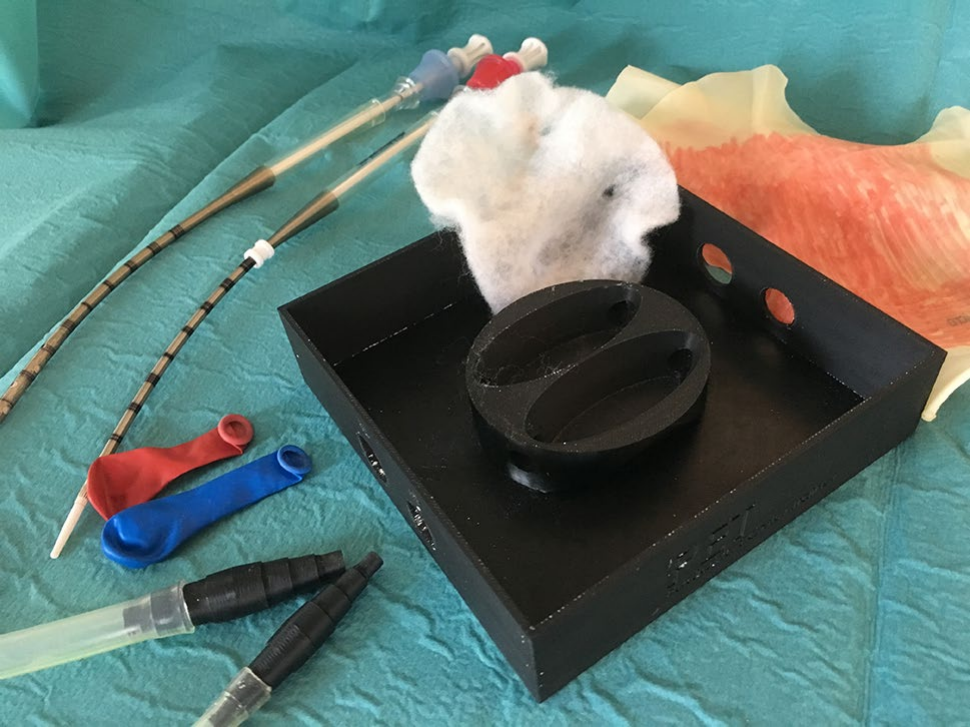
Separate components of the 3D model

**Fig. 2:**
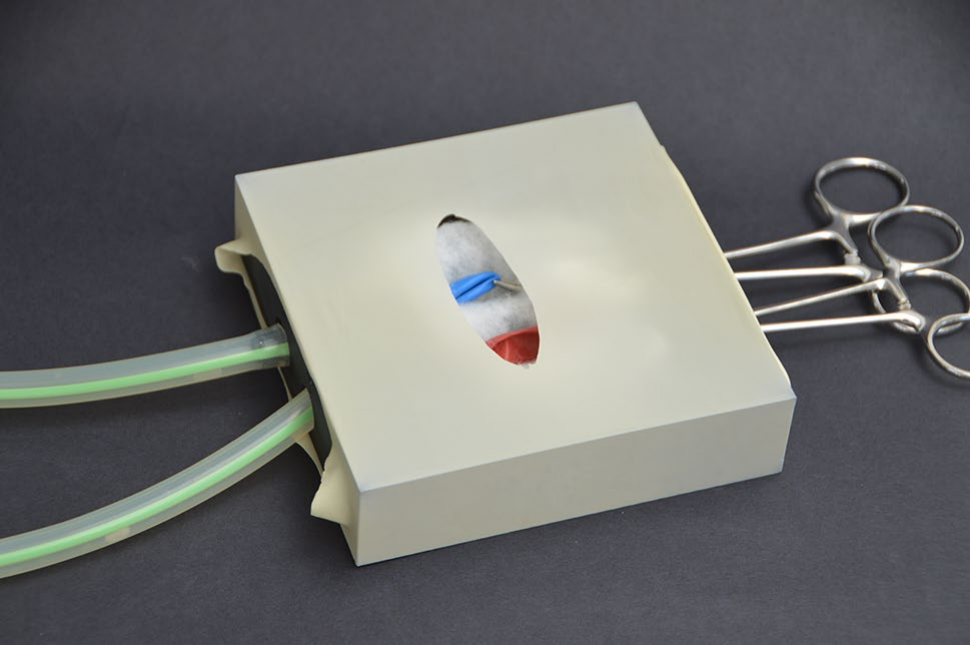
ECMO model after assembly

**Fig. 3:**
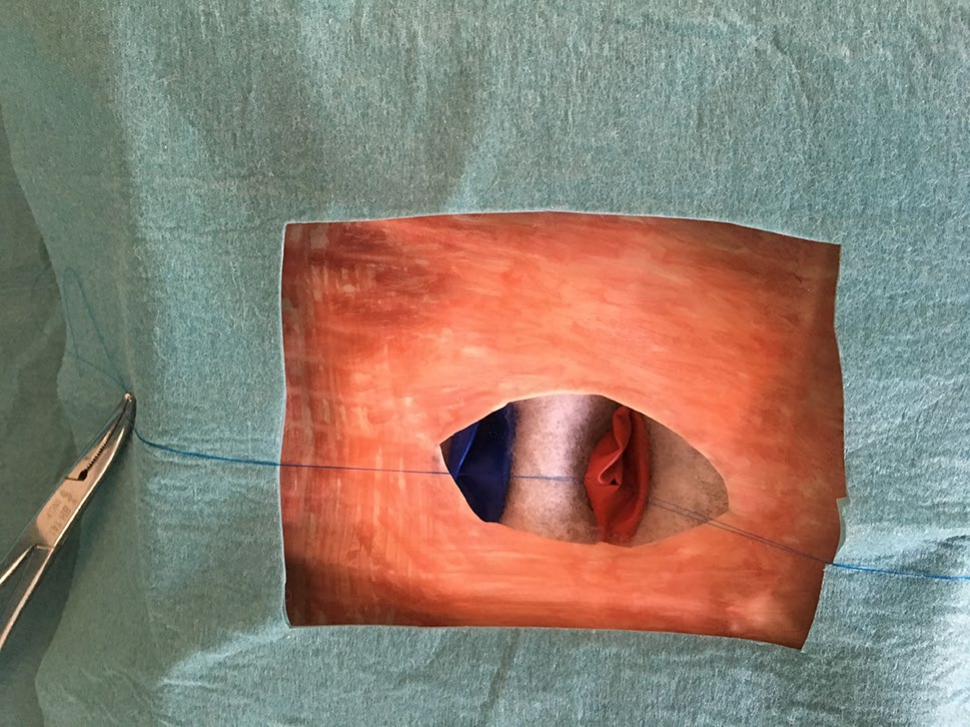
External view of model after assembly, after opening of the artery, with traction sutures

During the development process several models for the base were created using plaster. The model most resembling the clinical setting, was further developed to a 3D drawing and printed from plastic (Figs. 1, 2 and 3). The base and coupling pieces of the tubes were the 3D printed reusable parts of the model. To simulate the vessels, small water-balloons were used which were attached to the coupling pieces and held in place, through holes in the model, by small surgical clamps. The white layer (simulating the surrounding tissue) was made of foam bandage, to have a softer underground for the vessels, during both procedures of securing the vessels and inserting the cannulas in the vessels. The coupling pieces were developed to give some resistance during the insertion, especially for the arterial cannula. This was to simulate the snug positioning of the catheter in the vessels as in the clinical setting. The current model was specifically designed for 8 Fr arterial and 10 Fr venous cannulas, to ensure the snug fit and feeling of realistic resistance during the cannulations. For the arterial vessel, there was the option to use double layered balloons, to practice the avoidance of a dissection, which is one of the pitfalls of ECMO cannulation. The model was covered with a surgical glove, colored in a skin tone, to resemble the already opened skin. The subcutaneous tissue was not simulated in the model, because the dissection of the vessels was not part of the steps in this study. Dissection of vessels is difficult to simulate and requires a different development approach, which is not the aim of this low budget, reusable model.

### Training of procedure on the model

**Fig. 4:**
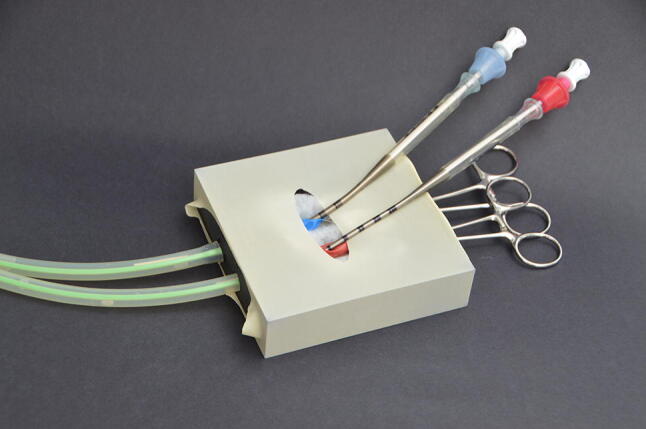
ECMO cannulas inserted in the ECMO model

**Fig. 5:**
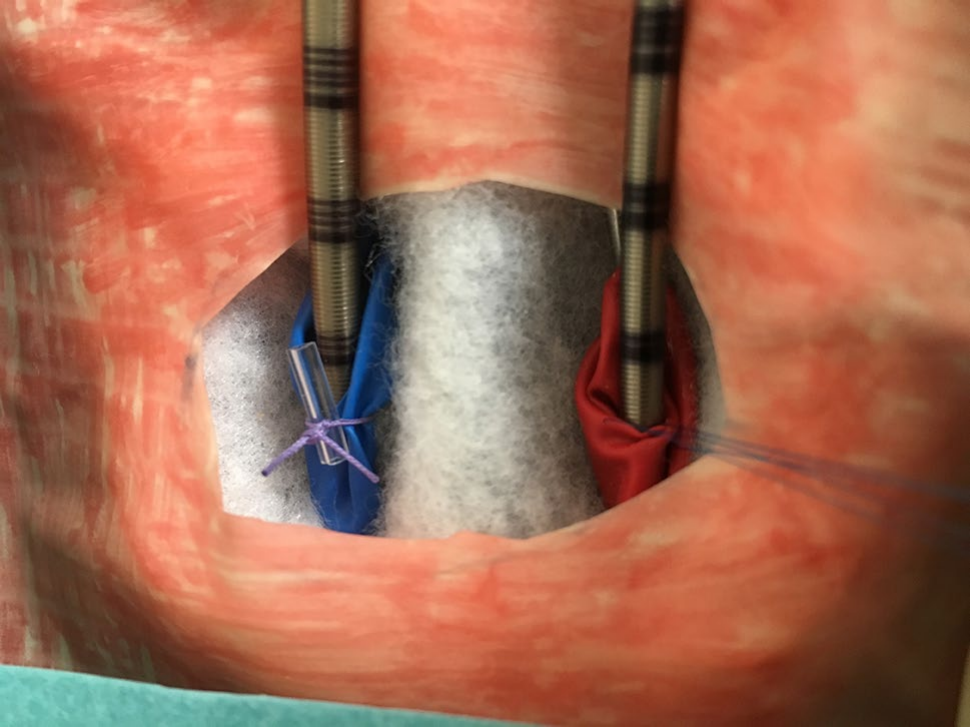
Close-up view of the cannulas in the simulated vessels

The cannulas have to be placed in the proper position, inside the tubes (Figs. 3, 4 and 5).

The following component steps of the ECMO cannulation can be performed on the model:

1. Securing the vessels.
2. Opening of the vessels.
3. Inserting the cannulas in the vessels.
4. Fixation of cannulas.
5. Connecting the cannulas to ECMO system.

The connection of the cannulas to an ECMO system can be simulated by connecting the tubes to an ECMO simulator or infuse system, to simulate blood flow through the vessels.

### Protocol

All participants were asked to train in the above mentioned steps on the ECMO model, to gain insight into the properties of the model and possible training applications. The first four steps were easy to train; however, because of limited options to connect the cannulas to an ECMO machine, the participants received instructions on how to do this. This was in the same manner as during normal cannulation, although it was not possible to have a higher flow than 50 ml/min, due to leakage. For training purposes it was not necessary to connect the model to the ECMO simulator or machine which made it easy to take it to the participants and use during training courses.

After the training process, the participants completed a questionnaire regarding their opinion of the model. All participants signed an informed consent declaration for the study, after which all data was processed anonymously.

### Participants

The participants were recruited during several national pediatric surgery courses, from March 2017 to November 2018, in the Netherlands and the 11th Pediatric Colorectal Congress, Nijmegen the Netherlands, 6–8th December 2018. The subjects were divided into two groups based on their self-reported experience: ‘target group’ with clinical pediatric surgery experience and had performed less five ECMO procedures themselves, consisting of pediatric surgery residents, fellows and young pediatric surgeons, because these are the intended users of the training tool The ‘experts’ had performed at least five to ten ECMO procedures themselves and assisted in at least an equal number.

### Questionnaire

The questionnaire was adapted from a previously used questionnaire in other validation studies [8–11]. It consisted of two parts, with the first part containing the consent, demographics and the participant’s current pediatric surgical experience. The pediatric surgical experience was defined as current profession/training level and number of ECMO procedures performed/supervised and assisted. The second part of the questionnaire consisted of questions on the realism of the model (regarding realistic response and tissue behaviour) and the specific component steps that could be trained on the model. This refers to the face validity of the model. The questions were rated on a five-point-Likert scale, in which ‘1’ means in strong disagreement, ‘3’ being the neutral opinion and ‘5’ meaning a strong agreement.

### Statistical analysis

The analysis was performed using Statistical Package for Social Sciences (SPSS) version 22 (IBM Corp., Armonk, NY). The results of the questionnaires were inserted into the database and the results were compared between the expertise groups, using an independent test. A *p* value of < 0.05 was considered a statistically significant difference in opinion between the expert and target group participants. To calculate the ratings compared to a neutral value (3.0 on the five-point-Likert scale) a one sample *t*-test was used. A mean of > 3.5 was considered a significantly better opinion than neutral, although a mean of 4.0 or higher was considered a powerful training tool for that component step.

## Results

In total, twenty-one participants completed the questionnaire after training on the ECMO model. There were fourteen experts and seven target group participants. Eleven were male, eight were female and two did not answer this question on the form. The mean age of the target group was 39 years and 49 years for the experienced pediatric surgeons. There were nineteen pediatric surgeons and two residents included in this study. The demographics and clinical ECMO experience of the total group and expertise groups are described in Table 1.

**Table 1:** Demographics and clinical experience of the participants in this study

Demographics	Total group (*n* = 21)	Expert group (*n* = 14)	Target group (*n* = 7)
Male:female (*n*)	11:8*	7:5*	4:3
Age (mean years, SD)*	45.3 (10.1)	48.9 (8.8)	39.0 (9.7)
Profession (*n*)			
Pediatric surgeon	19	14	5
Resident	2	0	2
Clinical experience (*n*)			
ECMO procedures performed			
None	5	0	5
< 5	1	0	1
5–10	3	2	1
11–25	3	3	0
> 25	9	9	0
ECMO procedures assisted			
None	3	0	3
< 5	4	1	3
5–10	4	3	1
11–25	3	3	0
> 25	7	7	0

*Two participants in the experienced group did not state their sexes

Table 2 describes the opinion of the participants of the model and component steps trained on the model. The only significant difference in opinion between the expertise groups was in the opening of the vessels, which was rated as realistic by the experts with a 4,0 on the five point Likert scale and a 3.4 by the target group (*p* = 0.032). There was also a difference of opinion on the possibility to connect the cannulas to the ECMO machine, in which the experts rated this better again (4.0 versus 3.5), although this was not seen as significant (*p* = 0.105).

**Table 2:** The grading of the items, in mean and standard deviation, which is based on a 5-point Likert scale (1: very bad, 3: neutral, 5: very good)

Opinion of the ECMO model	Total group	Target group	Expert group	*p*-value
	*n* = 21	*n* = 7	*n* = 14	
Visual aspects	3.8 (0.54)	3.9 (0.38)	3.7 (0.61)	0.519
Haptics of the landscape	3.6 (0.75)	3.7 (0.95)	3.5 (0.65)	0.605
Haptics of the vessels	4.0 (0.63)	4.0 (0.58)	4.0 (0.68)	1.000
Step 1: control of the vessels	3.8 (0.60)	3.9 (0.69)	3.8 (0.58)	0.818
Step 2: opening of the vessels	3.8 (0.51)	3.4 (0.54)	4.0 (0.40)	**0.032**
Step 3: placement of the cannulas	4.0 (0.78)	4.0 (0.58)	4.0 (0.88)	1.000
Step 4: fixation of the cannulas	4.0 (0.59)	4.0 (0.82)	3.9 (0.48)	0.836
Step 5: connection of cannulas	3.8 (0.62)	3.5 (0.55)	4.0 (0.60)	0.105

The significant differences between the target and expert group were calculated with the independent *t*-test

When comparing the opinion of the whole group with a neutral opinion, they were all statistically better than 3.0, with a *p* < 0.001 for all items, except the haptic aspects of the landscape, which was significant with a *p*-value of 0.002. When comparing the opinion with a value of 3.5, to consider these items as potent factors of the model, all items were significant, except the haptics of the landscape. The properties of the vessels were all rated significantly better than 3.5, (means 3.8–4.0, *p* = 0.002, *p* = 0.029 and *p* = 0.012), as were the placement and fixation of the cannulas (means 4.0, *p* = 0.008 and *p* = 0.002).

## Discussion

This low budget 3D printed model is reusable, with the aid of water-balloons, and therefore easy to use in preclinical training in any setting. It can be used in training programs, but more importantly it can be used at home or in the hospital to keep the structure of the steps of the cannulation up to date for all surgeons. ECMO cannulation can be a stressful and acute procedure, in which there is no time to prepare the procedure beforehand. Therefore, it is extremely important that these skills are optimized for any surgeon, at any time. Traditionally, cannulation training relies on the apprenticeship model, which could take a long time to overcome individual learning curves, particularly in lower volume centers. This model, however, can be used by anyone because the water-balloons can be changed after every practice. Video guidance will be provided with the model, when it becomes available for purchase (www.pediatrickBoxx.com), to aid the training without the need of an expert supervisor.

The model is considered to be realistic enough to train the component steps of the procedure by all participants. It is even considered more realistic by the expert than the target group. This could be based on the fact that the experts acknowledge the importance of the structured approach in this procedure and acute setting, while the target group often still focuses on the appearance of a model. The dissection of the vessels was not attempted to be simulated, because this would not be possible in a low cost, reusable model. However, the opening of the vessels still scored good (mean 3.8 by total group, which was significantly better than neutral, *p* < 0.001), especially by the experts (mean 4.0, significantly better than by the target group participants Table 2).

The establishment of a safe and effective ECMO program requires the presence of providers with adequate technical and team skills to cannulate infants and neonates in a timely fashion. Although preclinical surgical skills training has been shown to improve technical skills in the clinical environment [12, 13], such training does not give the surgeons explicit experience in handling the additional stressors of the operating room [14, 15]. It has been demonstrated that time pressure, noise, and other distractions impair dexterity and increase errors [16, 17]. However, surgeons, with more experience and technical skills, are better able to manage these stressors [17]. Fully contextualized cannulation training, though intuitively attractive, has been limited by the availability of adequate integrated trainers [7]. The model developed in the current study can be implemented in full training programs because it is possible to connect it to an ECMO simulator machine. Therefore, in full team training, it will be possible to add stressors to the cannulation training, after the steps of the procedure are mastered.

A previously described cannulation model was used in an ECMO training session, with good results [7], although the opinion on this model was only given by trainees and not by experts. They did show the added value of training the procedure on a preclinical model. However, they only performed two cannulations in this training, while previous studies showed a learning curve of at least 8–25 sessions to reach a plateau phase in the preclinical training [18–20], depending on the procedure, technique and training method. Even though the ECMO cannulation will be performed by a surgeon, with experience in other procedures, the particular steps of the cannulation are unique to this procedure. Therefore, more research is needed on the learning curve of this procedure to establish the optimal training sessions. It is also recognized that the skills or steps of a complex procedure will reduce over time, if these are not trained regularly (once a month) [21]. This model is a good tool to keep this structure up to date for all surgeons dealing with ECMO patients, particularly if they do not perform the procedure monthly.

The limitations of this model are the fact that it can be connected to an ECMO machine, but it cannot run on normal rotation speed yet, because of leakage. This was mainly due to the smaller cannulas (8 French and 10 French) and the large resistance of the flow. The 3D printed plastic was not strong enough to withstand this flow, but this is possible to overcome when using larger diameter cannulas. Because of the haptic feedback in the model it is important that the connecting pieces between the tube and balloon are snug around the cannula, therefore the higher flow will only be possible with an adapted model for > 12 French cannulas.

During this study, the model itself was not fixed to the table, therefore it moved easily because it was made from lightweight materials. Therefore, we have placed suction cups underneath it to fix it to the table to enhance the training session.

The reason for the relatively low number of participants giving their opinion, compared to other simulation models validated, is the low experience of surgeons with neonatal or infant ECMO. In the Netherlands, only two centers perform ECMO in children. Also, the aim was to use the real target group for this procedure, therefore only young pediatric surgeons and two residents were included.

Future studies should focus on the use of this model in a training curriculum, with long term training and assessment, to enhance the skills of the surgeons and improve the ECMO cannulation procedure.

## Conclusion

We have developed an easy to use 3D model for the training of ECMO cannulation, because training of this high risk procedure could reduce complications. Most component tasks of the procedure can be trained including securing of the cannulas to the vessels and connection to an ECMO system. This low budget model is considered a valid tool to train the component steps of the ECMO cannulation, which could avoid the learning curve in a stressful, clinical setting.

## Abbreviation

ECMO: Extra-corporeal membrane oxygenation
